# The interface of gambling and cultural practices: a Tongan male perspective in Aotearoa | New Zealand

**DOI:** 10.3389/fsoc.2023.1116312

**Published:** 2023-06-07

**Authors:** Edmond S. Fehoko, Maria E. Bellringer, Peggy Fairbairn-Dunlop

**Affiliations:** ^1^Division of Sciences, Department of Human Nutrition, University of Otago, Dunedin, New Zealand; ^2^Gambling and Addictions Research Centre, Faculty of Health and Environmental Sciences, Auckland University of Technology, Auckland, New Zealand; ^3^School of Social Sciences and Public Policy, Faculty of Culture and Society, Auckland University of Technology, Auckland, New Zealand

**Keywords:** Tongan gambling, cultural practices, obligations and responsibilities, talanoa, status advancement, Tongan social structure

## Abstract

**Introduction:**

For over 30 years, Pacific people have been identified as more at risk of developing problem gambling behaviors than the general population. That observation has not changed despite the increase in treatment service providers, Pacific gambling literature and problem-gambling literature, which are primarily quantitative-based. This article explores the interface of gambling and cultural practices from a Tongan male perspective to consider whether status advancement and rank contribute to the problem-gambling statistics and the qualitative reasons why Tongan peoples engage in gambling activities.

**Methods:**

A phenomenological approach using the talanoa research method was employed to carry out this study. A total of 46 Tongan males participated in this study. Interpretative Phenomenological Analysis was employed to interpret the data. The ethical approval reference number 16/452 for this research was granted by the Auckland University of Technology Ethics Committee (AUTEC).

**Results:**

Participants noted the concept of fatongia as a motivating factor for Tongans to engage in gambling activities, which, in turn, elevates family and village status and rank.

**Discussion:**

Strategies and recommendations around raising cultural awareness with treatment providers are critical to understanding Tongan gambling in New Zealand.

## 1. Introduction

Pacific peoples are at a greater risk of developing problem-gambling behaviors than any other ethnic group in New Zealand (Abbott and Volberg, 1991, 2001; Ministry of Health, 2008; Bellringer et al., 2013, 2017; Urale et al., 2015; Thimasarn-Anwar et al., 2017). While it is not clear why this is so, earlier studies on Pacific people reported that Pacific children are exposed to gambling at a very young age (Perese and Faleafa, 2000; Bellringer et al., 2013). Perese (2009) proposed that this could be because Pacific peoples may change their gambling behaviors if they migrate from where gambling was mostly illegal and opportunities were scarce to places where gambling opportunities were abundant. Gambling activities are illegal in Tonga and other Pacific nations. However, migrating to diasporic countries where gambling activities are readily accessible and available, such as New Zealand, entices Tongan people to engage in such activities to try to win and make money (Guttenbeil-Po'uhila et al., 2004). This supports findings with Asian immigrants in the United States of America, who have similar experiences (Welte et al., 2006).

In 2004, there was a concern raised by public health workers, their networks, and some Pacific community leaders about the significant number of Tongans experiencing the hazards of gambling (Guttenbeil-Po'uhila et al., 2004). Guttenbeil-Po'uhila et al. (2004) noted some unexplained patterns within the Tongan community which adversely affected their Tongan culture. As a result, stories linking gambling not only to financial loss but also to a range of health and social problems, including family and partner abuse, neglect of children and elders, and lack of supervision of young people, were circulating in the close-knit Pacific communities (Guttenbeil-Po'uhila et al., 2004).

Guttenbeil-Po'uhila et al. (2004) indicated that Tongan males would gamble at Totalisator Agency Board (TAB) outlets to escape the isolation experienced upon migration to New Zealand. These findings were corroborated by Clarke et al. (2007), who indicated, in their review of the literature, that several factors such as “social isolation, disconnectedness, boredom, socio-cultural ambivalence, financial hardship, under-employment, and the need to participate in acceptable recreational activities” have been identified as triggering factors for problematic gambling amongst migrant groups. Guttenbeil-Po'uhila et al. (2004) found that trying to meet and carry out cultural obligations and responsibilities contributed to why Tongans would gamble.

As a result of their gambling behavior and financial hardship, Tongans could not meet monetary obligations such as church donations and children's school fees (SHORE/Whāriki MasseyUniversity, 2004). There were reports that Tongan loan outlets had very loose credit criteria and would lend on more lenient terms so that, in some cases, credit checks were not a requirement, which suited clients who already had bad credit ratings (Guttenbeil-Po'uhila et al., 2004). Furthermore, Tongan pawnbrokers and small personal loan outlets have also seen significant growth in clientele and business. Personal home loans have also been set up to target this dubious market (Guttenbeil-Po'uhila et al., 2004). Research has also reported the pawning of Tongan cultural goods because of gambling (Guttenbeil-Po'uhila et al., 2004; Centre for Social Health Outcomes Research Evaluation Te Ropu Whāriki, 2008).

## 2. Tongan cultural practices

Tongan people place a high value on life events, and associated social occasions such as birthdays, graduations, weddings, and funerals (Morton-Lee, 1996; Ketu'u, 2014). To that point, the following tables capture the average economic cost of cultural and monetary gifts to key family members, church ministers and distinguished guests.

Milestone birthdays—birthdays celebrated at the ages of 1, 16, 21, 50, and 70 onwards—are particularly important occasions for Tongans (see Table 1). These birthday celebrations are determined by the family status in the church or community, which, in turn, determines the quantity and quality of the koloa faka-Tonga^1^ (Tongan mats and crafts) presented to key people, including the fahu (father's sister), church minister, distinguished guests (that is, nobles, royal family members, and members of parliament). While the average cost may seem high in monetary value, this is outweighed by the faka'apa'apa (respect), 'ofa (love), tauhi vaha'a (maintaining relationships) and fetokoni'aki (reciprocal respect) that is shown to the family. During these birthday celebrations, individuals, friends, and family often offer a dance, cultural mats or monetary gifts to show appreciation. It is important to note that Tongan families are now spending less than the average total cost mentioned in the table due to the exclusion of the cultural exchange of gifts and crafts, and the availability of catering services.^2^

**Table 1:** Average economic cost of a Tongan birthday celebration.

**Cultural process**	**Cultural items**	**Cultural purpose**	**Monetary value for each item (NZD)**	**Total cost (NZD)**
Presentation to Fahu	1. Fala tekumi ma nima 2. Ngatu Launima 3. Kato teu monomono fakapalangi 4. ‘Umu	Tapa cloth mats and food gifted to the matriarch of the family	1. $1,500.00 2. $3,000.00 3. $400.00 4. $300.00	$5,300.00
Presentation to the Faifekau	1. Kie Tonga fute hongofulu 2. Ngatu Launima 3. Sila 4. ‘Umu	Mats, food and money gifted to the religious minister	1. $800.00 2. $3,000.00 3. $1,000.00 4. $400.00	$5,200.00
Presentation to a distinguished guest	1. Konga kie fute hongofulu 2. Fala paongo tahanima 3. Sila 4. ‘Umu	Mats, food and money gifted to distinguished guests i.e. individual from Royal Family	1. $300.00 2. $800.00 3. $500.00 4. $400.00	$2,500.00
Individual dance	1. Tekiteki 2. Ngatu Launima 3. Fihu Fatufa	Mats and monetary gifts from individual dances were gifted to distinguished guests.	1. $1,000.00 2. $1,500.00 3. $2,500.00	$6,500.00
Tongan style feast for 150–200 guests	1.30 roasted pigs @$170.00 each 2. Food and beverages	The quantity of food is important to the success of the occasion.	1. $5,100.00 2. $10,000.00	$15,100.00
Total average economic cost				$34,600.00

The average economic cost of a Tongan funeral excludes the burial plot,^3^ funeral homes fees, vehicle hire and other services,^4^ coffins and other non-cultural necessities (see Table 2). Furthermore, this economic cost can fluctuate depending on the deceased's level of social status in the family or community. For example, the cost will increase to a much higher level if the deceased is from the nobility, royal family, or educated elite, or was a government minister or from a wealthy family (James, 1996; Ketu'u, 2014).

**Table 2:** Average economic cost of a Tongan funeral.

**Cultural process**	**Cultural items**	**Monetary value for each item (NZD)**	**Total cost (NZD)**
Mourning period until family members arrive	1. Dinner and supper for family members per night $15.00 pp—Evening prayer (50 people) for five nights	1. $3,750.00	$3,750.00
Night of the wake/Day of the burial	1. Dinner per person (approx. 350 people) @ $25.00 per person	1. $8,750.00	$8,750.00
Distribution of food and koloa faka-Tonga	1. Cultural mats for the fahu 2. Two kilograms of raw meat per person (400 people @ $20.00 per person) 3. Cultural mats and monetary donation to church ministers (average 6 ministers @ $500.00 per minister).	1. $3,000.00 2. $8,000.00 3. $6,000.00	$17,000.00
Presentation to the fahu	1. Cultural mats 2. 1 large-sized pig	1. $3,000.00 2. $500.00	$3,500.00
Three nights of mourning	1. Food per night—evening prayer for three nights (average of 80 people @ $15.00 pp) 2. Gift for the lead preacher per night @$300.00 per night	1. $3,600.00 2. $900.00	$4,500.00
Pongipongi Tapu	1. Food for those who attend (average of 150 people @ $20.00 pp) 2. Maumau to the fahu	1. $3,000.00 2. $1,500.00	$4,500.00
Total average economic cost			$42,000.00

As highlighted in the table below (see Table 3), a typical Tongan wedding takes almost a week to complete, and includes the following phases:

(i) Faitohi: Formal proposal by the groom to the bride's parents to marry their daughter. This is regarded as the most respectful way of honoring the bride and her family. Furthermore, families exchange multiple cultural gifts, food, and drinks. The barter between talking chiefs for the bride and groom is also showcased for the night.
(ii) Fakalēlea: This is the pre-wedding evening, two nights before the actual wedding day where both families exchange multiple cultural gifts, food, and drinks. The barter between talking chiefs for the bride and groom is also showcased for the night.
(iii) Kātoanga mali: This phase is the actual wedding day which includes the wedding ceremony followed by a grand feast.
(iv) ‘Ave ‘o e mohenga: The night of the actual wedding day, the bride's family also take her belongings, which often include a queen-sized bed, dining tables and lounge suites, to the groom's home.
(v) ‘Uluaki Sāpate: Important to Tongan people during the wedding is the first Sunday service ceremony followed by a feast with family and church members.
(vi) Fai ‘o e ‘api: This phase is where the newly-wed couple consummates their wedding, and the bedsheet is expected to be stained with blood if the bride is a virgin.
(vii) ‘Ave ‘o e ‘api: Once the wedding is consummated, if the white sheet is stained with blood, it is then taken with cultural mats to prove the bride's virginity.
(viii) Tali ‘o e ‘api: The stained white sheet is then taken to the bride's mother with cultural mats, food and beverages to acknowledge the bride, her mother and the wider family. The last three stages are intended to honor the bride and show respect to her parents for remaining loyal and faithful to her husband-to-be.

**Table 3:** The average economic cost of a Tongan wedding.

**Cultural process**	**Cultural items**	**Monetary value for each item (NZD)**	**Total cost (NZD)**
Faitohi: Tongan Proposal	1. Fala uangokumi 2. Ngatu fuatanga 3. Ngatu launima 4. 20 cartons of soft drinks 5. 40 cakes 6. 1 large-sized pig	1. $1,800.00 2. $600.00 3. $3,400.00 4. $600.00 5. $800.00 6. $400.00	$7,600.00
Fakalēlea	1. Fala uangokumi 2. Ngatu fuatanga 3. Ngatu launima 4. 20 cartons of soft drinks 5. 40 cakes 6. 1 large-sized pig	1. $1,800.00 2. $600.00 3. $3,400.00 4. $600.00 5. $800.00 6. $400.00	$7,600.00
Kātoanga Mali: Actual Wedding Day	1. Fala uangokumi 2. Ngatu launima 3. Lau tefuhi 4. Fihu fatufa 5. 30 roasted pigs @$170.00 each 6. Food and beverages	1. $1,800.00 2. $3,400.00 3. $1,500.00 4. $1.800.00 5. $5,100.00 6. $12,500.00	$26,100.00
‘Uluaki Sāpate: First Sunday	1.10 roasted pigs @$170.00 each 2. Food and beverages	1. $1,700.00 2. $5,000.00	$6,700.00
**‘Ave ‘o e mohenga: Taking her bed and necessities**			
**Fai ‘o e ‘Api: Consummating the Wedding**			
‘Ave ‘o e ‘Api: Taking the White Sheet to her Mother	1. Me'a hina 2. Falauanoa 3. 1 medium-sized pig 4. Food and beverages	1. $500.00 2. $1,800.00 3. $200.00 4. $150.00	$2,650.00
**Tali ‘o e ‘Api: accepting the consummation**			
Total average economic cost			$50,650.00

Similar to Table 2, these economic costs do not include the traditional costs of the wedding, such as the suits for the groom and his groomsmen,^5^ rings,^6^ cakes,^7^ transport for the bride and groom,^8^ and the wedding venues.^9^ Furthermore, the economic costs of a Tongan wedding listed in Table 3 are only for the groom and exclude the costs incurred by the bride and her family.

The three tables above are compelling evidence of the average economic cost of a Tongan birthday, funeral, and wedding. As a result, Tongan people may often resort to gambling to meet such cultural demands (Perese and Faleafa, 2000; Guttenbeil-Po'uhila et al., 2004; Urale et al., 2015).

According to Lātukefu (1974, p. 173), fatongia was formerly used to describe the enforced labor of commoners for chiefs. Interestingly, despite the abolition of commoners' fatongia in the 1862 Code of Laws, and the restraint of chiefly powers and privileges by the constitution, considerable chiefly demands upon commoners continue to the present day (Lātukefu, 1974; Morton-Lee, 2004). Fatongia refers explicitly to the duties involved in social relations, mainly providing services and food to higher-status people and kāinga members (Morton-Lee, 2004, p. 92). Furthermore, fatongia is often seen as reciprocal, so those of higher status also have obligations to those of lower status. As scholars put it, the ideology of reciprocal obligation represents “high” and “low” people as upholding an ideal of mutual dependency, sacrifice, and service motivated by warm emotion and loyalty (Lātukefu, 1974; Biersack, 1990). Like the Tongan culture, the concept of fatongia is constantly evolving, adapting and changing with every generation (Tu'itahi, 2005; Vaioleti, 2011; Tanaki, 2015). Similar to the concept of fa'alavelave in the Samoan way of life, fatongia is seen across Pacific gambling literature as a cultural practice which drives gambling activities for the raising of funds among Pacific communities. Thus, this article explores the Tongan male perspectives on the intersection of gambling and cultural practices in New Zealand.

## 3. This study

This article explores the interface of gambling and culture from a Tongan youth perspective. The research design was interpretivist/constructivist and phenomenological, as understood through the lens of a Tongan worldview. Participants comprised of two groups—elders born in Tonga who migrated to New Zealand, and New Zealand-born Tongan youth. Participants were recruited through snowball sampling from churches, kava-drinking circles and other community spaces. A total of 28 Mātu'a (elders) (see Tables 4, 5) and 18 To'utupu (youth) (see Tables 6, 7) participated through focus group talanoa (FGT). Talanoa is to share without concealment in individual engagement and focus group discussions—(see Churchward, 1959; Vaka et al., 2016). The importance of the talanoa method is grounded on the notion of nurturing relationships with participants before and after the data collection. Furthermore, the use of the mother tongue language of the participant either on documents or in individual and focus group discussions.

**Table 4:** The Mātu'a age groups—focus group Talanoa.

**Participants**	**Ages**	**Participants**	**Ages**
Mātu'a 1.1	60s	Mātu'a 2.1	40s
Mātu'a 1.2	50s	Mātu'a 2.2	60s
Mātu'a 1.3	50s	Mātu'a 2.3	40s
Mātu'a 1.4	60s	Mātu'a 2.4	40s
Mātu'a 1.5	50s	Mātu'a 2.5	50s
Mātu'a 1.6	40s	Mātu'a 2.6	50s
Mātu'a 1.7	40s	Mātu'a 2.7	70s
Mātu'a 1.8	70s	Mātu'a 2.8	60s
Mātu'a 1.9	50s	Mātu'a 2.9	40s
Mātu'a 1.10	40s	Mātu”a 2.10	40s
		Mātu'a 2.11	70s
		Mātu'a 2.12	50s

**Table 5:** The Mātu'a age groups—individual Talanoa.

**Participants**	**Ages**
Ma'ake	40s
Maika	40s
Mote	50s
Misi	60s
Mone	60s
Miu	60s

**Table 6:** The To'utupu age groups—focus group Talanoa.

**Participants**	**Ages**	**Participants**	**Ages**
To'utupu 1.1	30s	To'utupu 2.1	20s
To'utupu 1.2	20s	To'utupu 2.2	20s
To'utupu 1.3	20s	To'utupu 2.3	18-20
To'utupu 1.4	20s	To'utupu 2.4	30s
		To'utupu 2.5	20s
		To'utupu 2.6	20s
		To'utupu 2.7	18-20
		To'utupu 2.8	20s

**Table 7:** The To'utupu age groups—individual Talanoa.

**Participants**	**Ages**
Tevita	20s
Toni	30s
Taani	20s
Taniela	20s
Tui	30s
Tika	20s

Pseudonyms were used for all participants in the FGT and individual talanoa. Pseudonyms with numbers were participants from the focus groups. Pseudonyms with names were participants from the individua talanoa. Informed consent were given also given for the FGT and individual talanoa. These talanoa were in Tongan or English, as appropriate, and audio-recorded and transcribed by the first author. As a result, this study employed a descriptive thematic analysis approach while drawing on components of Interpretive Phenomenological Analysis to analyse the data from the Mātu'a and To'utupu FGT and individual talanoa. Thematic analysis provided a highly flexible approach, enabling a rich, detailed and complex understanding of the talanoa data to be gained (Braun and Clarke, 2006). As Braun and Clarke (2006) argued, thematic analysis is a valuable method for examining the perspectives of different research participants, highlighting similarities and differences, and generating unanticipated insights. Braun and Clarke (2006) state that thematic analysis reports the participants' experiences, meanings and reality. Thematic analysis was also valid for summarizing key insights and views shared in the FGT and individual talanoa. It gave the first author a well-structured approach to considering and handling these experiences and perceptions.

## 4. Results

Most of the To'utupu pointed to their distinct cultural responsibilities and obligations, such as satisfying cultural expectations, to explain why Tongan males gamble. While fundraising activities had aspects of gambling, some spoke about how socio-cultural responsibilities and obligations such as extravagant 16th and 21st birthdays and weddings may also propel Tongan individuals and families into financial pressures to meet specific cultural standards. This view aligns with the Pacific gambling literature, which suggests that the need to meet cultural demands contributes to Pacific people participating in gambling. For example, Toni argued the view that gambling will eventually be normalized as part of the Tongan way of life.

I strongly believe it is going to become a norm. Gambling will become, if not already, a part of our culture. It is going to become a norm where everyone is going to accept it and people will be like, it is not hurting you nor we so just let be. Take raffle tickets for example, it is another form [of] gambling because it involves money. Raffle tickets has already become a norm in the Tongan culture where our people think it is a quick fix for raising money. [Toni]

Similar views were shared by several of the Mātu'a. For example, one participant commented on how bingo has become commonplace in the Pacific.

Like for the Samoans and Cook Islanders, bingo or housie has become a norm you know, even before coming to New Zealand. Gambling is going to go from an unspoken event to a more spoken event but do not talk about it because it has been around for years. [Mātu'a 1.3]

In another example, several To'utupu participants argued that Tongan people engaged in gambling with the hope of trying to win a lot of money which, in turn, will increase family status in the community. One said that Tongans would gamble not only to fulfill socio-cultural responsibilities and obligations but also to win a significant amount of money that would increase the status and rank of their family and village.

I would also say that culture contributes because of the pressure to provide, the pressure to be seen that we [Tongan families] can stand on our own two feet. I had seen 21st birthdays and seen people and families struggle to meet the cultural demands. [To'utupu 2.5]

Some of the Mātu'a commented on how participating in the polies can also be viewed as a gambling activity where additional money can be gained in an effort to meet their cultural goods and responsibilities. For example, as Miu commented:

So, they would go to the pokie machines or casinos for a quick fix to get that increase. So, I questioned why they even had the celebration and all these expensive stuff like the koloa faka-Tonga [Tongan tapa and mats] stuff when you cannot even afford it you know, it is frustrating! You know, at the weekend, I was at a birthday because they hired me to take photos, and so we had already set up all the paperwork, and I am cheap compared to the professionals, and then they turned around to me and said, “we cannot pay you”, and I was like, people are throwing money left, right, up and down and so right now, I am holding photos from my customers unless they pay up—what doesn't make sense is that they could throw a good birthday party at a fantastic venue and yet cannot pay me, rubbish. [Miu]

Toni also said that the collective nature of Tongan family life influenced the decision-making of those engaging in gambling. Many referred to how they had always been taught to share whatever they had, whether this is food, money or other goods. As Toni suggested, sharing is in our nature (in Tongan culture); if one wins, the kāinga also wins.

Like with the Tongan culture, we supposedly love and share with people where we would always share or vahevahe our money. So, when he wins, it is not just him winning but the entire kāinga winning as well because they would feel entitled to get a share as well. [Toni]

There is an expectation that Tongan males are the major breadwinners who ensure families' basic needs are met and families' cultural and social responsibilities to the kāinga are fulfilled. In the FGT, To'utupu 1.1 said that the responsibilities and duties of Tongan males in New Zealand still play an influential role in Tongan society. The majority of the To'utupu proposed that if Tongan males do not supply the kāinga, they fail to meet the needs of their families, church and culture.

Pacific Island males, a majority of them are breadwinners and so knowing that they would take that money [to gamble] to get a growth out of it and when they know there is not much to supply to the family, whatever fatongia for the family, church or what is on you know. [To'utupu 1.1]

Several of the Mātu'a also referred to the cultural expectations that Pacific males endured. For example, the majority of the Mātu'a reflected on their jobs and agreed that gambling can become an alternative to generating additional income to meet family needs.

There is an expectation but there is also the responsibility that comes along being a Pacific male. You know as a male, you had to supply for your family and if you cannot, then you do not consider yourself as a male, you would consider yourself as someone who does not live up to the expectations that their forefathers was able to do. [Ma'ake]

For majority of the Mātu'a and To'utupu, they acknowledged that Tongan values such as faka'apa 'apa (respect) encourage Tongan people engage in gambling behaviors. For example, several participants commented on how money has now become a motivating factor to elevate an individual and family status in the community. Thus, one participant reflected on how one family's status changed with gambling winnings.

I remember this family at church won almost a million dollars and the very next day they prepared a grand feast for the church. Throughout that church service, the church respected this family differently, looked at differently all because of their winnings from the casino. [Matu'a 2.6]

This section explores the cultural factors and challenges that motivates Tongan people to engage in gambling activities to try win money to fulfill cultural responsibilities, which in turn, elevates one's status in Tongan families and communities.

## 5. Discussion

While gambling appears to be found in almost all cultures and most parts of the world, there has been a significant gap in the gambling literature regarding the role of culture in gambling and problem gambling (Custer and Milt, 1985; Raylu and Oei, 2004) and, significantly, in understanding when cultural beliefs and values can influence gambling behaviors and help-seeking attitudes (Subramaniam et al., 2015). Culture plays a vital role in gambling. Cultural beliefs and values can influence gambling behaviors and help-seeking attitudes (Raylu and Oei, 2004). Research has found that people from cultures with values and beliefs that favor gambling are more likely to gamble or develop problem gambling than those from cultures that do not have values that encourage gambling (Raylu and Oei, 2002, 2004). Similarly, people from cultures with negative attitudes toward getting professional help are less likely to try to get help when they initially begin experiencing problems with their gambling. Thus, they are more likely to continue gambling and subsequently develop problem gambling (Subramaniam et al., 2015).

Binde (2005) suggested that a sense of honor and prestige was recognized in acquiring gambling winnings across many ethnic cultures. Guttenbeil-Po'uhila et al. (2004) also reported that gambling was often pursued to increase family and social status. Interestingly, the majority of the To'utupu put forward the idea that underpinning Tongan gambling is the desire for Tongan people to elevate their familial status and rank. With that, several Mātu'a suggested that gambling, notably winning considerable amounts of money from gambling, may elevate the individual and familial status and rank. Similarly, some To'utupu posited that when Tongans elevate their individual and familial status and rank in Tongan society, they also seek the same respect given to nobles and high chiefs. This is a key issue for Tongan people in Auckland, but one that is under-recognized as an issue by Tongan communities, government, and social services alike. Also, the majority of Tongan people reside in low socioeconomic areas and low-income households (Statistics New Zealand, 2018), which also indicates why Tongans and other Pacific peoples view gambling activities as an opportunity to generate additional income. Further in-depth research is needed to explore this matter.

There are also negative behaviors associated with how Tongans practice these core cultural values (Ritterbush, 1986; van der Grijp, 1993). Some of the more significant behaviors include fesiosiofaki or envy, ngutulau or gossip, fakamā or shame, and fakavahavaha'a or competition. For example, such behaviors sometimes drive an individual, family or village to give large amounts of money during a mission or church donation ceremony, birthday, funeral or wedding, rather than a genuine willingness to help (Ketu'u, 2014). Thus, Tongan people often resort to gambling to meet the cultural demand of changing perceptions and status.

Perese and Faleafa (2000), who conducted the first Pacific-specific ethnic gambling study in New Zealand, found that the Samoan culture and religious beliefs forbid gambling participation. Yet, gambling is acceptable and seen as a “rite of passage” in New Zealand (Perese and Faleafa, 2000). Culturally grounded perceptions about gambling were held more particularly by the Mātu'a who, in a sense, had justified a habit of gambling behaviors in the past. For example, gambling has been associated with recreation, socializing, competition and pride in winning. Not only that, but the terms also used to describe these games of chance suggested these activities were insignificant pastimes.

Furthermore, monetary contributions to the family, church or community from gambling were indicated by several To'utupu as a respectful way of elevating the individual and familial status and rank (Perese, 2009). Faka'apa'apa (respect) as a practice value also has implications for such matters. For example, significant monetary contributions to family, church or community events such as weddings, funerals and birthdays elevate the status and rank of individuals, families and villages. As reflected by several of the Mātu'a and To'utupu, the practice of faka'apa'apa underpins the desire to make significant monetary contributions to such events to change the perceptions of other Tongan individuals, families and villages. Consequently, gambling is perceived as a vehicle to win additional money to make such contributions. The cultural core values of sharing, reciprocity, respect and maintaining good relationships are essential as they help maintain society's unity and harmony. However, some participants reported that their practices needed to be scaled down, given the financial burdens they place on individuals, families and communities in Tonga and abroad (Ketu'u, 2014).

## 6. Limitations

There are a number of limitations in this study. First, this study does not represent the total population of Tongan males in Aotearoa | New Zealand. A longitudinal study is needed to explore the intergenerational transmission of gambling harm. Second, all participants were recruited from one city within in Aotearoa | New Zealand. Further study to capture gambling and problem gambling behaviors in other cities across Aotearoa | New Zealand is needed.

Theoretically informed gender analysis for gambling harm is rare (Palmer du Preez et al., 2021). This study, focused as it is on a male perspective, had the opportunity to explicitly explore gender related issues, notions and practices influence men's gambling in this context. There was some exploration—men's socio-cultural positioning as primary “breadwinners”—but not enough to extrapolate possibilities for gender-responsive and gender-aware harm prevention and reduction activities. A deeper understanding of masculine assumptions, practices, expressions, and implied values could have been illuminated (although the sample was quite small). More engagement with indicative of gendered social and cultural discourses and processes shaping their practices and experiences.

## 7. Conclusion

This article has explored the interface between gambling and cultural practices in New Zealand from a Tongan male perspective. The role of culture in gambling behaviors is an emerging field of inquiry. For Tongan and Pacific peoples in New Zealand, upholding cultural values, beliefs and practices is central to one's cultural identity and belonging outside the homelands. Thus, it is no surprise that gambling was mainly conducted to gain sufficient funds to meet, maintain and re-affirm their standing and social status within the Tongan family, church and community. There is a need for future research to fully understand the relationship and influence between gambling and culture in ethnic and Indigenous communities. Moreover, more is needed to design more effective harm minimization initiatives and culturally targeted awareness campaigns.

## Data availability statement

The raw data supporting the conclusions of this article will be made available by the authors, without undue reservation.

## Ethics statement

The studies involving human participants were reviewed and approved by Auckland University of Technology Ethics Committee. The patients/participants provided their written informed consent to participate in this study.

## Author contributions

EF led the writing of the manuscript and data collection of the study. All authors were involved in the study design, contributed to the interpretation of the results and analysis, which was reviewed by all authors who approved the final version, and read and approved the final manuscript.

## Footnotes

^1^Koloa faka-Tonga is an overarching term for textiles made by Tongan women. The practice takes many forms, including ngatu, a cloth normally made from bark and inscribed with intricate patterns and symbols in dark inks; ta‘ovala, mats woven from strips of panadanus leaves. Certain koloa are saved for one-off ceremonial occasions and then stowed away, while others like the ta‘ovala are worn multiple times a week.

^2^The average cost for Pacific catering services is $25.00 to $40.00 per person.

^3^The recent update on a burial plot in the memorial garden is $4,287.00 (Auckland City Council, 2019).

^4^After reviewing the funeral packages in Auckland, the average cost for funeral services including transport, transfers, embalming and treatment, and coffin and death certificate is around $3,500.00.

^5^The cost of a suit ranges from $800.00–$1,500.00. With that, the average number in a groomsmen's line ranges from 3 to 10, with a cost of approximately $5,000.00–$10,000.00.

^6^On average, a groom's wedding ring ranges from $800.00–$2,000.00.

^7^Average number of cakes in a Tongan wedding ranges from 3 to 10 cakes, with each cake consisting of mats, ‘umu and, at times, money.

^8^Depending on the groom's preference, the cost of vehicles could range from free to $1,200.00 for the day.

^9^The average cost of a wedding venue outside the church ranges from $2,000.00 to $20,000.00, and this cost often includes catering services.
